# Supplementary material for: The Impact of Defect Size on Bone Healing in Critical-Size Bone Defects Investigated on a Rat Femur Defect Model Comparing Two Treatment Methods
Source: Bioengineering (Basel). 2024 Mar 19;11(3):287. doi: 10.3390/bioengineering11030287 (PMC10968167; doi:10.3390/bioengineering11030287)
Supplement: Supplementary file 1 [file bioengineering-11-00287-s001.zip › bioengineering-2877363-supplementary.pdf]

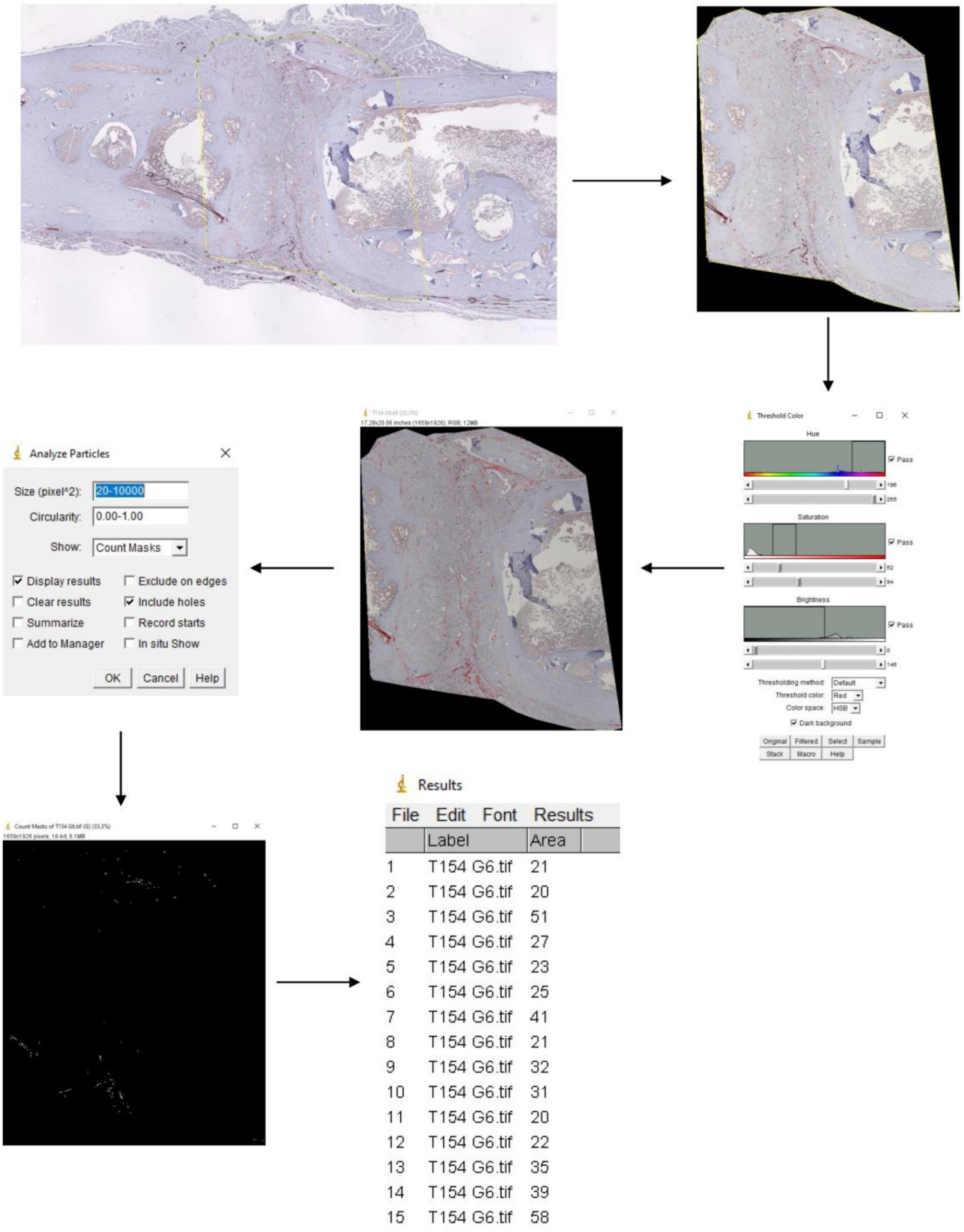

Supplemental Figure S1: Strategy for histomorphometric evaluation of blood vessel area fraction in the bone defect area using the software *ImageJ*. The defect area is marked (yellow line) using the *Polygon* tool and subsequently cropped from the surrounding tissue and its area is determined. Stained blood vessel structures were identified and marked in red by application of the *treshold color* tool. The *analyze particles* tool enables the setting of criteria to withdraw staining artifacts from evaluation. The *Count mask* displayed the vessel structures (white) whose areas were subsequently summarized.

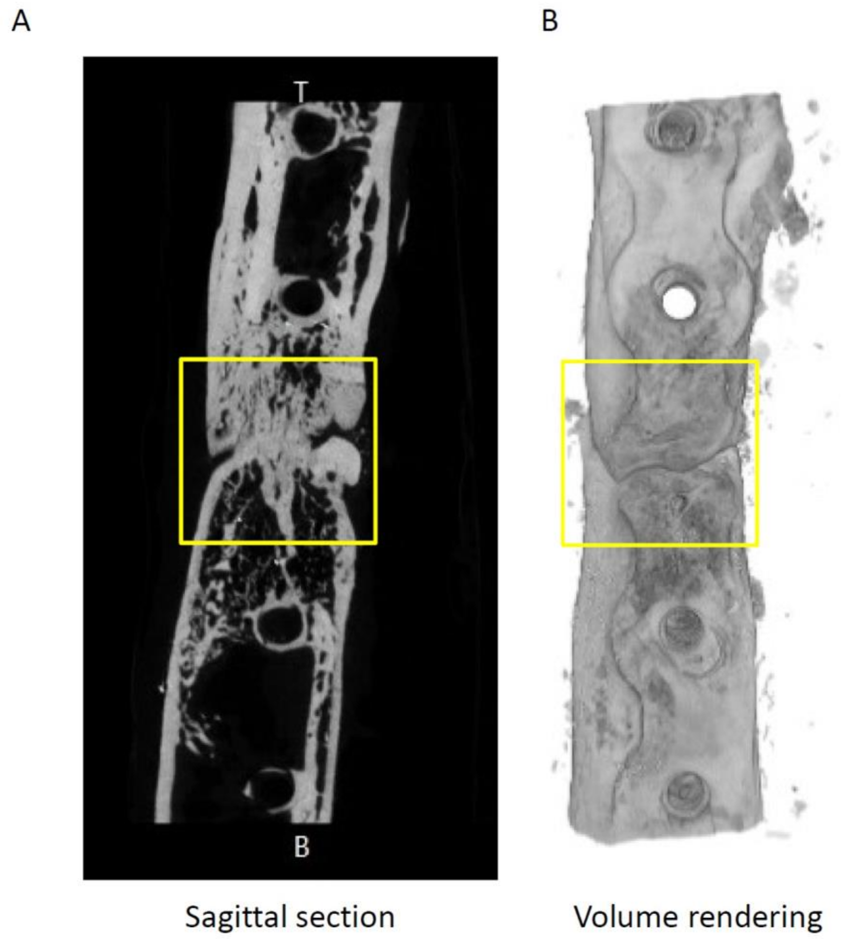

Supplemental Figure S2: Sagittal section (A) and volume rendering (B) of a rat femur computed on base of 2D µCT-image stack. A 5 mm bone defect treated with the induced membrane technique is shown. The region of interest (ROI) that was used for evaluation of BMD and BV/TV, is marked by the yellow square.
